# A predictive model for understanding the role of emotion for the formation of presence in virtual reality

**DOI:** 10.1371/journal.pone.0280390

**Published:** 2023-03-16

**Authors:** Crescent Jicol, Hoi Ying Cheng, Karin Petrini, Eamonn O’Neill

**Affiliations:** University of Bath, Bath, United Kingdom; University of Milan, ITALY

## Abstract

Users’ emotions may influence the formation of presence in virtual reality (VR). Users’ expectations, state of arousal and personality may also moderate the relationship between emotions and presence. An interoceptive predictive coding model of conscious presence (IPCM) considers presence as a product of the match between predictions of interoceptive emotional states and the actual states evoked by an experience (Seth et al. 2012). The present paper aims to test this model’s applicability to VR for both high-arousal and low-arousal emotions. The moderating effect of personality traits on the creation of presence is also investigated. Results show that user expectations about emotional states in VR have an impact on presence, however, expression of this relationship is moderated by the intensity of an emotion, with only high-arousal emotions showing an effect. Additionally, users’ personality traits moderated the relationship between emotions and presence. A refined model is proposed that predicts presence in VR by weighting emotions according to their level of arousal and by considering the impact of personality traits.

## 1 Introduction

In the past few decades, VR technology has evolved significantly in its technical capabilities, producing ever more realistic environments and increasingly sophisticated virtual worlds. We will specifically refer to VR as the head-mounted displays (HMDs) that are able to immerse users in virtual worlds. Presence has been described as the sense of “being there” [1], which suggests that it can be influenced by individual differences. The concept of presence can be operationalized either as a feeling or state of consciousness [2]. In the present work presence will be considered as a state of consciousness, in line with the definition given by Seth et al. [3]. Within the context of video games, higher presence seems to enhance user satisfaction, enjoyability and time spent playing [4, 5]. Heightened presence has also been shown to increase usability of VR as a tool in domains such as psychology research [6, 7], learning [8–10], training [11, 12] and therapy [13, 14], and in commercial advertising [15] and sensorimotor rehabilitation [16, 17]. Given its growing significance, it is important to develop a better understanding of presence, and how it may be created and harnessed to enhance the enjoyability, usability and effectiveness of VR applications.

This paper will thus focus on understanding the creation of presence in VR. For clarity, we distinguish between presence and immersion which, although related, are distinct concepts and therefore should not be used interchangeably [18, 19]. Slater and Wilbur [20] defined immersion as a characteristic of technology rather than a personal trait. In investigating the formation of presence in VR, research has often focused on the contribution of technical features [21], however, some previous work suggests that users’ emotions may make an even stronger contribution than technical features to the creation of presence [22–24]. Hence, presence has been recognized as a multidimensional construct [24, 25] and divided into three separate components, namely spatial, social and self-presence [26, 27]. Still, the role that different emotions play in the formation of these components of presence has not been tested. To further complicate the issue, some research suggests that not only felt emotions but also users’ pre-exposure expectations about emotional valence and intensity can moderate subsequent presence in VR [3]. Thus, we will operationalise emotions based on the circumplex model of core affect, as defined by Russell [28]. This proposes that emotions are characterised by two main dimensions, namely arousal, which ranges from high to low, and valence which also ranges from positive to negative. For example, happiness is an emotion characterised by high arousal and positive valence, while fear is also high in arousal but negative in valence. In contrast, relaxation is positive in valence but low in arousal. Lastly, personality traits such as openness to experience, agreeability and introversion have been shown to moderate presence [29]. Overall, the processes that lead to the formation of presence in VR remain little understood.

## 2 Background and related work

### 2.1 Types of presence

In previous studies, presence has often been regarded as a unitary variable defined as the sensation of ‘being there’ (see [1, 30]. This definition is related to spatial presence, which in the context of VR represents a state of mind characterised by an impression of being amongst landmarks present in a virtual environment (VE) [31]. Because the ability to assess distances and locate oneself relies largely on technical features, these features have often been the focus of research. Studies using 2D screens or VR head-mounted displays (HMDs) have generally shown that technical features such as increased update rate [32, 33], tracking [34], field of view [35], image quality [36], stereoscopy [37, 38] and higher quality of sound [39, 40] increase spatial presence.

However, technical features may not be the only factors to influence the formation of presence. The influence of non-technical, user based factors was posited by Lombard and Ditton [41] who argue that presence is the “perceptual illusion of non-mediation”. Thus, presence may be influenced by both technical and user based factors. To account for this range of factors, Lee et al. [26] and later Tamborini and Bowman [27] divided presence into three distinct but interrelated dimensions: spatial presence, social presence and self presence. As noted above, much of the existent research has been directed to the study of spatial presence.

Social presence refers to users perceiving that they share a space with other conscious beings whose thoughts or emotions they can perceive and with whom they may have dynamic interactions [27, 42, 43]. Social presence seems to be of great importance to user experience in VR [42, 44]. Social presence has been researched substantially in the past, with studies identifying factors ranging from technical to contextual and individual differences being identified as contributing to its formation (see systematic review by Oh et al., [44]). For example, a study by Shin [42] asked participants for subjective appraisals of elements which would make them perceive themselves as present in a VE. The majority referred to presence as the extent to which they could feel they were together with other avatars in the VE. Participants also commented on the importance of feeling connected with other social users present in the VE when referring to presence [42]. In a recent study, Riches et al. [45] showed that in cases where avatars behave in an intelligible way, or in which interaction with avatars is natural, overall presence is greatly enhanced. However, despite emotions having been shown to influence feelings of social connectedness in the real world [46], relatively few studies investigated the role played by emotion in the formation of social presence in VR. One such example is a study by Sajjadi et al. [47] who attributed emotional states to avatars within a VR environment and observed that this had an impact on user levels of social presence. This means that not only users’ felt emotions but also those of other avatars can contribute to the formation of social presence. Still, the underlying process by which social presence is created and the exact role played by emotions in it are not yet fully understood.

Compared to social presence, self presence remains relatively under researched. Cummings and Bailenson [48] excluded social and self presence from their meta-analysis of the effect of immersive technology on presence because there were “simply too few studies in the existing literature that empirically examine the effect of particular immersive features on social and self presence” (p.277). Self presence has been defined as the psychological state in which users regard their virtual self within the VE as their actual self [26, 45]. Biocca [43] takes this point further and talks about the impression of existing as a social being within the VE, which is an extreme form of self presence and is related to social presence. Similar to social presence, the process that determines self presence and the role played by emotion in this process are yet to be fully understood.

These three dimensions of presence—spatial, social and self—should not be regarded as unrelated. It would be expected that they coexist to some extent for presence to be achieved. For example, if the user achieves self presence but other avatars in the VE lack psychological involvement or behavioral interaction, overall presence would be jeopardized. Similarly, if the formation of spatial presence is hampered (e.g. due to a lack of afforded interactivity), this would render the VE less believable as a real space, and thus social and self presence may in turn be hampered.

### 2.2 Models of presence

Studies have often treated presence as a unitary concept, which may have contributed to the myriad of contrasting theories on the formation of presence. Descriptive models of presence focus on distinguishing the components of presence and put less emphasis on the actual process that creates presence, hence their name. Thus, descriptive models are heavily impacted by technological approaches that strive to improve VR through technical advances. Early evidence of this trend is exemplified by Schubert et al.’s [49] Igroup Presence Questionnaire (IPQ), which identified spatial presence, involvement and realness as the main components of presence. The most prominent descriptive model is the Process model on the formation of spatial presence, proposed by Wirth et al.’s [1].

This model predicts that spatial presence is facilitated by the existence, believability and accuracy of spatial cues present in a VE. This is in turn dependent on whether the user is able to perceive the VE as a plausible space in the first place.

Despite the useful contribution of descriptive models, structural models of presence may offer a better understanding of self presence and social presence because structural models focus on how presence is created in the mind. The user is considered a mediator between bodily interoceptive states and real world exteroceptive signals [50]. Exteroception refers to the perception of the environment via classical sensory modalities, i.e. visual, auditory, tactile etc. Interoception, on the other hand, is the perception of physiological conditions in one’s own body [3, 51]. People create expectations about the stimuli they will encounter, which produce physiological responses that they can perceive (interoception). These internal states are subsequently compared with the actual stimuli that are subsequently perceived (exteroception).

### 2.3 Interoceptive Predictive Coding Model (IPCM)

**Fig 1:**
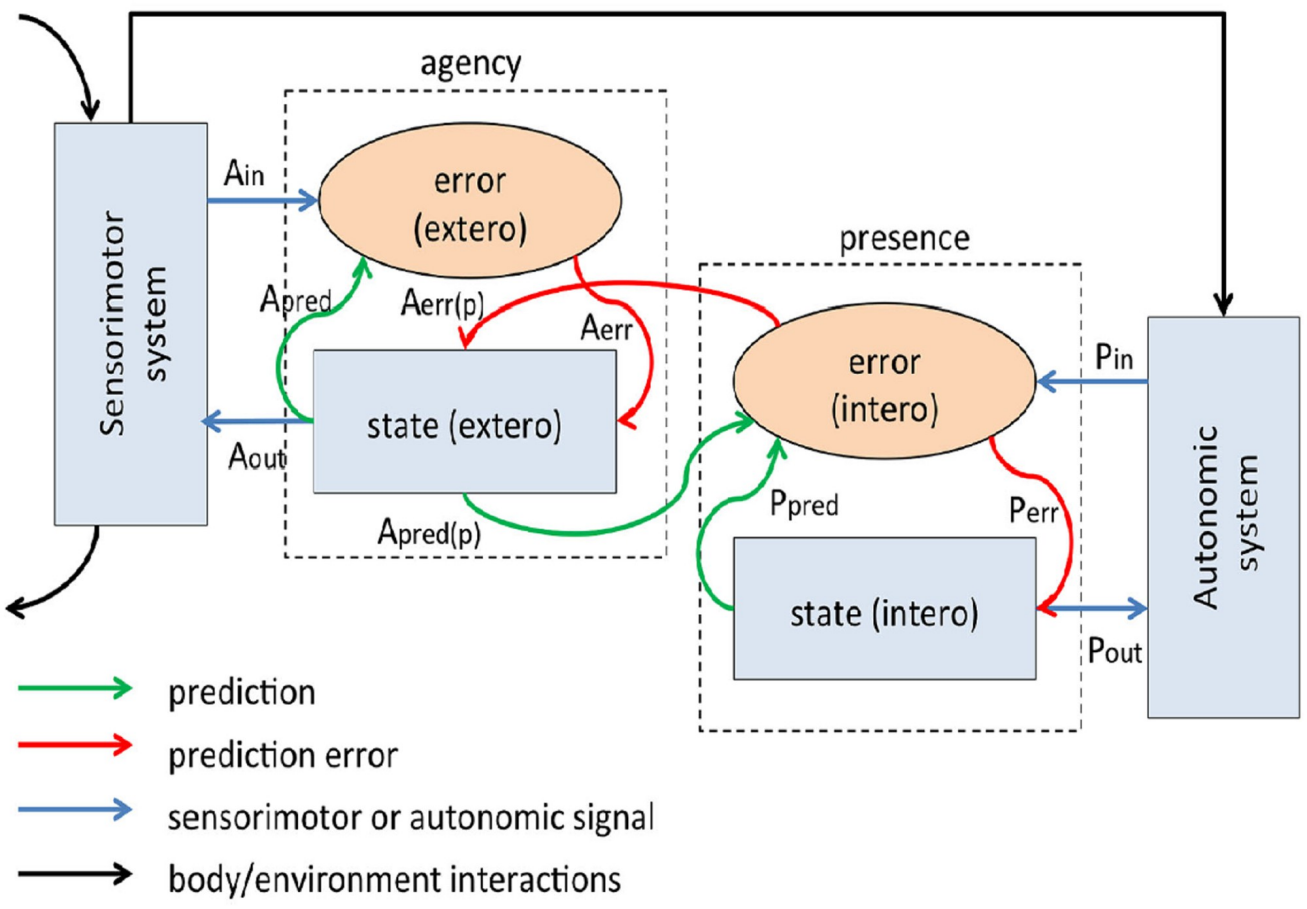
Figure from Seth et al. [3] that illustrates the fundamental architecture of the IPCM model. “Both agency and presence components comprise state and error units; state units generate control signals (Aout, Pout) and make predictions [Apred, Ppred, Apred(p)] about the consequent incoming signals (Ain, Pin); error units compare predictions with afferents, generating error signals [Aerr, Perr, Aerr(p)].

Based on this concept of interoception, Seth et al. [3] formulated the Interoceptive Predictive Coding Model (IPCM) of Conscious Presence. This structural model stipulates that presence felt in the real world or in a VE is a product of the matching between predictions of interoceptive (emotional) states and actual states created when encountering external stimuli. Thus, users predict the type and intensity of emotion that a certain experience would elicit and then match the felt emotion with this prediction. Although there will always be a degree of mismatch, the IPCM postulates that presence is the result of a good match between these two processes. If the mismatch is greater, in order to achieve presence the user must exert additional effort to suppress information from the external stimuli that is incompatible with their expectations (See Fig 1).

Applied to VR, the IPCM postulates that prior to experiencing a VE, the user would predict the emotions that the VE would elicit [3]. For example, before trying a scary VR experience, the user would predict high fear and low relaxation. This expectation would be accompanied by neurophysiological reactions in the user’s body [3]. If the intensity of the predicted fear is lower or higher than the actual fear felt during the VR experience, the user has to suppress this mismatch to achieve presence. This idea of suppression of expectations that are incompatible with the VR experience has been postulated before [50, 52]. Sheridan, in his estimation theory [52], talks about the necessity for continuous suppression of disbelief, which determines the formation of presence through a continuously updated interior model of the environment. However, the above-mentioned study focused on the prediction of events occurring in the environment [52]. In contrast, the IPCM emphasizes the prediction of internal states, thus placing the user at the centre of the presence formation process.

The IPCM is supported by neurophysiological and neuroimaging studies investigating the role of brain regions such as the anterior insular cortex (AIC). Most importantly, the AIC has been shown to be responsible for the comparison between predicted and actual interoceptive signals [53, 54]. This is what the IPCM describes as the process by which emotions contribute to the formation of presence. One study used VR to simulate a roller coaster experience while brain activity was measured using functional magnetic resonance imagery (fMRI). Several brain areas were activated more when participants reported strong presence, amongst which was the AIC, compared to when they did not, although this effect was only observed in a sample of children, but not adults. This partly supports the IPCM’s assumption that there exists a relationship between presence and predicted and felt emotions [3]. Moreover, hypoactivity of the AIC has been found in patients with depersonalization disorder (DPD). This condition is characterized through a loss of subjective sense of reality of self and of the world [55, 56]. This is further corroborating evidence that predictive coding may have an influence on presence. Nonetheless, more neuroimaging and neurophysiological studies are needed to clarify the exact role of emotion in the creation of presence at the neural level.

### 2.4 Presence and emotion

The relationship between presence and emotion has mostly been studied by means of correlations, so the directionality of any causation is impossible to derive [24]. For example, Baños et al. [24] exposed users to relaxing and joyful VR environments and found that presence and emotion intensity were positively correlated. Nonetheless, the authors acknowledge that this correlation does not elucidate whether higher presence leads to more intense emotions, or if this effect manifests in the opposite direction. Other experiments in VR exposure therapy have reported significant correlations between the level of presence and intensity of emotions [57–59]. According to Frijda’s [60] law of apparent reality, emotions are elicited in situations that are being perceived as real, and their intensity is directly proportional to the degree to which this is the case.

Several studies have assumed that presence drives emotion and not the other way around [50, 61, 62]. Still, some research points to an inverse effect; that is, emotions may drive presence. For example, while investigating the use of VR for treatment of arachnophobia, it was found that presence was enhanced when a spider was introduced in the VE [22]. A study by Baños et al. [23] independently manipulated technical features by presenting the same VE via an HMD, a computer monitor and a video wall. Emotional valence of the VE was also controlled: in one scenario it was sad while in the other neutral. Results showed an interaction effect between technical features of the system, presence and emotion. Technical features were found to affect presence only in the neutral condition and not in the emotion condition. Higher levels of presence were however found in the emotion condition, in line with previous research [22]. Similarly, VR has been used before as a distraction tool in patients with burn wounds, showing that it is more powerful than conventional media in reducing patient anxiety during painful medical procedures (see systematic review by Morris et al. [63]). Overall, these results suggest that emotional content could represent a stronger driver of presence than technical features.

### 2.5 Testing the IPCM

The IPCM, like other presence models, does not distinguish between different components of presence in VR, preferring to simplify it into a unitary concept. This is problematic because, as mentioned, predictive coding can be expected to apply mainly to social and self presence and less to the formation of spatial presence. There is a need to test how these components of presence are achieved individually and to what extent emotions and expectations contribute to their creation.

Secondly, emotions differ in characteristics such as arousal, with feelings of fear and anxiety characterised by high levels of arousal increasing with sense of presence [64] and emotions with low arousal such as relaxation not showing such an association to presence [24, 57]. This suggests that the relation between presence and emotion could be moderated by the type of emotion despite a similar level of arousal (e.g., happiness and fear) or by the level of arousal (e.g., fear and relaxation) [65].

Thirdly, distinct highly arousing emotions could manifest differently. Fear, unlike happiness, is an important evolutionary survival function that triggers fight or flight responses [66]. Due to its vital importance, fear can be elicited via two pathways at neural level: a perceptual (e.g. by seeing fear-inducing cues) and a conceptual pathway (e.g. information on an incoming fear-inducing stimulus). Perceptual fear-related cues are known to evoke instant physiological and behavioural reactions (e.g. seeing a snake). Similarly, conceptual information about fear-inducing stimuli can determine a weaker, yet significant physiological activation [67] (e.g. being informed that you will encounter a snake). This is supported by the Emotional Processing Theory which suggests that information about an imminent stimulus (priming) pertaining to an emotion in itself induces that emotion to an extent [68]. This process is also the basis for the prediction and subsequent matching of emotions on which the IPCM is based. In the present paper we will refer to such information provided to participants as priming. However, it remains unclear whether such effects would be present for other emotions such as happiness which, although arousing, do not have such strong importance for survival. To this end, the applicability of the IPCM will be tested with both fear and happiness. As a further comparison, the model will also be tested with relaxation as it is a low-arousal emotion that is well documented [24, 50].

Finally, the IPCM does not account for individual differences derived from personality traits. It has been shown that personality traits such as neuroticism, conscientiousness and extraversion have a significant moderating effect on emotion regulation, notably suppression [58]. In addition, it seems that personality traits play a decisive role in how users engage with factors determining presence [32, 35, 69–71]. For example, willingness to try, openness to new experiences and introversion are important factors which may moderate presence within a VE [29].

## 3 Research questions and hypotheses

It is apparent that presence as a construct is more complex than previously thought, and the exact role of emotions, expectations and personality traits for the formation of different components of presence remains to be explained. Arousal may moderate this process and so also may prior perceptual or conceptual priming. The potential moderating effect of personality factors also bears investigation. To address these points, four research questions were formulated.

**Research Question 1:** Does the IPCM predict the different types of presence (spatial, social and self presence) in VR?

**Research Question 2:** Are different emotions affecting presence differently in VR?

**Research Question 3:** Do conceptual and perceptual processes lead to different levels of presence?

**Research Question 4:** Do personality traits moderate the relationship between emotion and presence?

The IPCM treats presence as a unitary concept but, as discussed, it can be more informative to treat its components separately. Because the impact of emotion is expected to be more prominent in social and self-presence, we predict the IPCM to apply in their cases but not in the case of spatial presence. As discussed above, we would expect high-arousal emotions to have a more pronounced effect on presence compared to low-arousal emotions. In addition, within highly-arousing emotions, fear should have the most pronounced effect due to its evolutionary survival role. As argued above, perceptual priming should elicit stronger emotions compared to conceptual priming, because actually encountering a stimulus elicits a stronger response than information about said stimulus [67]. We might therefore expect perceptual priming to have a larger effect than conceptual priming on the relationship between emotion and presence. In regard to the moderating effect of personality traits, previous research suggests that openness, introversion and agreeability could have an impact on presence. Thus, we predict that these traits will act as moderators between emotion and presence. Hence, we have the following experimental hypotheses.

**Hypothesis 1:** We predict that smaller mismatches between predicted and felt emotion (IV) will lead to higher levels of social and self presence, but not spatial presence (DV).

**Hypothesis 2:** We predict that highly-arousing emotions (fear, happiness) will have a more pronounced effect than low-arousal emotions (relaxation) (IV) on social presence and self-presence (DV). We expect fear to have the most pronounced effect of all.

**Hypothesis 3:** We predict that perceptual priming (IV) will affect presence (DV) more than conceptual priming (IV), and we expect this effect to be strongest for fear.

**Hypothesis 4:** We predict that the personality traits of openness, introversion and agreeability (IV) will moderate the relationship between emotion and presence (DV).

## 4 Method

### 4.1 Experimental design

The experiment adopted a between groups design with seven participant groups. Three groups were primed with perceptual stimuli (PP) while another three received conceptual priming (CP). The seventh group received no priming and acted as the control group. Thus, the seven experimental conditions were as follows: PP + relaxation, PP + happiness, PP + fear, CP + relaxation, CP + happiness, CP + fear and the control group. From hereon we will distinguish between ‘predicted’ emotions, referring to the intensity of a given emotion that the user expected they would feel within the VR experience prior to being exposed to it, and ‘felt’ emotions, which represent the retrospective subjective appreciation of the intensity of emotions they felt in said VE. Within the experimental design, the predictor variables were the type of priming (conceptual or perceptual) and the scores for predicted and felt emotions (relaxation/happiness/fear). Additional predictor variables were computed as differences between predicted emotion scores and felt emotion scores for the three emotions. The five personality traits measured via the Big Five Inventory (BFI) were also used as predictors [72]. The outcome variables were spatial presence, social presence and self-presence, measured via subjective questionnaires, plus two overall presence measures used and standardized by previous literature [73, 74]. The two measures assessing overall presence were the UCL Presence Questionnaire [73] and item number two from a questionnaire used by Egan [74]. The study took place in the VR/motion capture lab at the University of [REDACTED].

### 4.2 Emotion measures

In order to test the IPCM, we computed scores from predicted and felt emotion intensity scores. Thus, predicted and felt emotion intensities had to be assessed in a similar manner and on a similar scale. We opted to collect self-report measures of emotion rather than, for example, physiological measures because the IPCM is based on subjective expectations of emotions. Previous studies such as that of Riva et al. [75] successfully utilised concise measures of emotion consisting of one question. Similarly, a recent VR study investigating the role of emotions in VR presence formation has utilised the same emotion measures (see. [76]). Thus, we replicated Riva et al.’s [75] questions assessing participant levels of relaxation, happiness and fear and used three items. We chose a 1–10 scale for the emotion questions because they were administered verbally by the experimenter and it was previously shown that a 10-point scale is easiest to respond verbally to [77]. The same scale was used by Baños et al. [23] in their study where they also assessed participant emotion within VR.

The questions measuring emotions felt during the VR experience were phrased as: ‘For the following emotions, how intensely did you feel them during the VR experience, 10 being the highest and 1 being the lowest?’. Below the question, relaxation, happiness and fear were listed, each with a scroll bar set by default in the middle of the scale, at 5. The scroll bar could be dragged to indicate the level of emotion felt. To measure predicted emotions before VR the same questions were rephrased: (‘Rate how intensely you think you will feel the following emotions after the VR experience, 10 being the highest and 1 being the lowest’). The items about emotion predictions were administered so as to later compute the difference between predicted and felt emotions.

### 4.3 Presence measures

To assess presence, we used a battery of questionnaires. We administered the UCL Presence Questionnaire as it is a well-established measure of presence [73]. It also has been successfully used in investigating the relation between emotion and presence in VR [75]. In addition, three custom items were created to assess spatial presence, social presence and self-presence respectively, as described by Lee et al. [26]. Thirdly, an item from Egan et al.’s [74] presence questionnaire was used since this study also correlated presence with emotional states. This item was the only one in Egan et al.’s questionnaire which referred to levels of presence. We used this range of measures of presence because previous studies have shown that correlations of presence with personality traits vary greatly depending on the questionnaire being used [78]. By using multiple measures we intended to verify whether results would indeed be inconsistent as past research would suggest. All presence questionnaires, including our custom one, were marked on a 7 point rating scale, which is what the authors proposing them suggested [73, 74]. From here onward our three custom measures of presence will be referred to as spatial, social and self-presence. For simplicity and clarity, we will refer to the UCL Presence Questionnaire as UCL Presence. Because it was used by Egan [74] as the sole measure for presence, we will refer to the second question from the questionnaire used by them as Overall Presence (see Table 1). In this study, none of the measures mentioned above were combined. Instead, we will assess and discuss them separately. As with measuring emotions, questionnaire measures were also preferred to physiological measures of presence because the latter have produced contradictory results in several studies [74, 79, 80].

**Table 1:** The UCL presence questionnaire [73], the custom items assessing spatial, social and self-presence and the second question (Q2) from Egan and colleagues [74].

Item	Question Text
**UCL Q1**	Rate your sense of being in the virtual environment.
**UCL Q2**	To what extent were there times during the experience when the virtual environment was reality for you?
**UCL Q3**	When you think back to the experience, to what extent do you think of the virtual environment more as somewhere that you visited than images that you saw?
**Spatial Presence**	I felt physically present in the virtual environment.
**Social Presence**	I felt like other characters (avatars) were actually present with me within the environment.
**Self-Presence**	I felt that the events were actually happening to me.
**Q2 from Egan et al. (Overall presence)**	I did not feel a strong sense of presence whilst experiencing the system.

### 4.4 Personality measure

The Big Five Inventory (BFI) which contains 44 items [72] was used to measure participants’ personality traits. This was chosen because it was used in previous studies to investigate the effect of personality traits on emotion suppression and regulation [58], which may have an important effect in the presence creation process [3, 81]. Additionally, Kober [78, 82] used the BFI and showed that it correlates with presence in VR. Other studies investigating personality of participants in non-VR computer mediated environments have also used the BFI successfully [29, 83–85]. Five main personality traits were investigated: extraversion *vs* introversion, agreeableness *vs* antagonism, conscientiousness *vs* lack of direction, neuroticism *vs* emotional stability, and openness *vs* closedness to experience.

### 4.5 Audio files

For the perceptual priming conditions, three pieces of classical music were chosen for their ability to induce the three desired emotions. For relaxation, Chopin’s ‘Nocturne, op. 15: 6 in G minor’ was chosen [86]. For happiness, Beethoven’s ‘Symphony No. 6, 3rd movement’ was chosen [87, 88]. Lastly, Holst’s ‘Mars, the bringer of War, op. 32: 1’ from ‘The Planets’ was selected to induce fear [89]. Each piece was played for 90 seconds, similarly to other studies that induced emotions using the same pieces [87, 88].

For the conceptual priming conditions, three ‘reviews’ were written about the VR experience we used, portraying it respectively as relaxing, happy or fearful. The text of the three reviews was kept the same but key words related to emotions were changed accordingly (e.g. fearful to relaxing; unfriendly to friendly). The reviews were pilot tested on a sample of five people who estimated that the reviews reflected similar levels of relaxation, happiness and fear respectively. The reviews were then read aloud by a female voice and recorded using a high quality microphone. This was done to ensure that the music and reviews were presented via the same sensory modality, i.e. auditory.

### 4.6 Apparatus

An Oculus Rift HMD was used for presenting the VR environment. This headset has a maximum refresh rate of 90Hz and uses OLED panels with a pixel density of 1080x1200 per eye. To enhance sound quality, a pair of Sennheiser HD 380 Pro headphones was used instead of the built in Oculus Rift headphones. The HMD was powered by an Alienware Area 51 desktop computer running Windows 10, with a 3.4 GHz Intel Core i7, 16 gigabytes of RAM, and an Nvidia 1080 Ti graphics card with 11 gigabytes of GDDR5 memory.

### 4.7 Virtual reality experience

**Fig 2:**
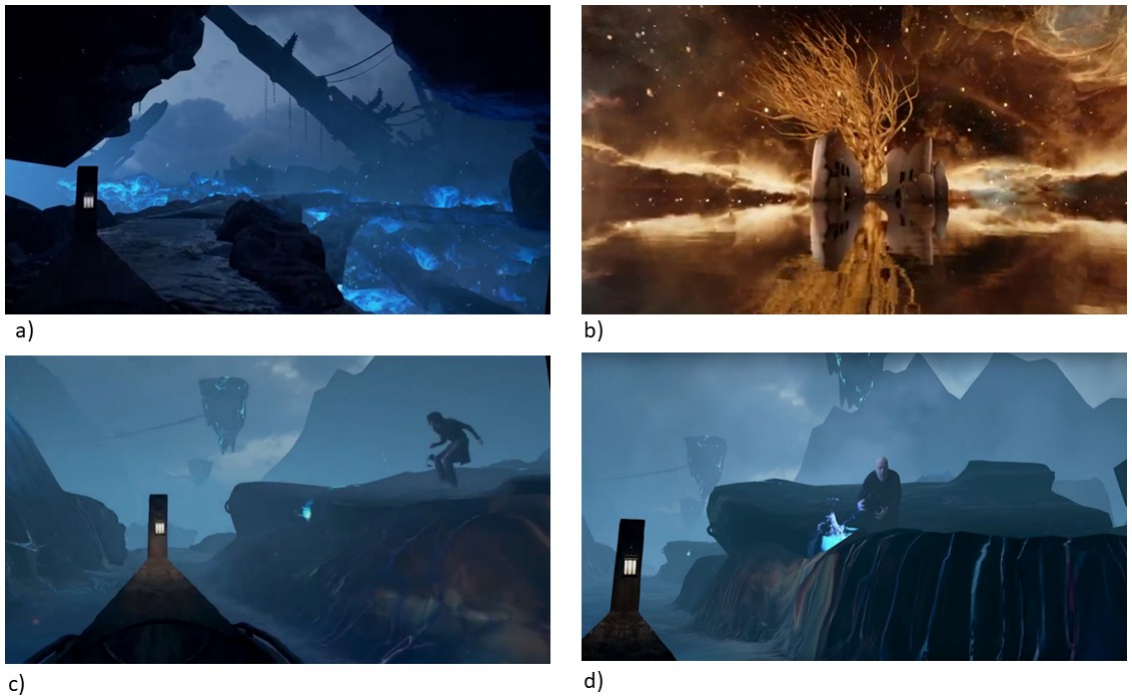
Screenshots taken from the user’s perspective in the VE. The experience presented a surreal journey in a Venetian gondola. Figure b) shows a starry sky mirrored in a lake on which the gondola is floating. Figures a, c and d) show the gondola at various stages of the journey. The full experience is a VR version of a mini-opera by Cory Strassburger and Alain Vasquez [92]. It is available free from the Oculus Store [90].

A VR experience from the Oculus Store named ‘Senza Peso’ [90] (see Fig 2) was chosen. This is an Italian opera-like experience dominated by intense visuals and vocals. Participants see themselves in a Venetian gondola that floats along a canal, surrounded by a surreal environment populated by characters placed on either side of the canal. Along the journey covered by the gondola, there are several environments which are visually stimulating and include fire and water cascades (see Fig 2). Additionally, several characters appear on the side of the water at certain times and they are performing actions such as searching with a lantern, or lighting a fire. This was also important since it was intended for the VE to elicit social presence as well. ‘Senza Peso’ was piloted together with a range of other candidate VR experiences prior to the current study with 36 participants (nine males, 27 females) over the age of 18 (mean age = 21, range = 18–44). They were asked to rate elicited emotions. ‘Senza Peso’ was interpreted by equal numbers of participants as relaxing, happy or fearful and it was therefore chosen for the main study in order to control for one emotion being evoked more compared to the others, which could have biased our results.

The experience lasted for approximately 7 minutes. This has been shown to be the ideal duration for a passive VR experience as the level of presence is expected to reach maximum levels around the 7 minute mark, after which boredom can ensue [19]. Because assessment of presence was done after the experience, the seven minute timing was important so as to avoid a recency effect whereby participants would report boredom although they had reported high presence during the experience. In order not to add agency as a potentially confounding factor, no active input or interaction by the participant was facilitated. The experience facilitated six degrees of freedom (three-axis rotational tracking plus three-axis positional tracking) for head movements. Participants did not have a perceptible body in the experience as this would have introduced new confounding variables in the experimental design (see [91]).

### 4.8 Procedure

Participants were met by the experimenter and were asked to read the information sheet and complete the consent form. Participants were randomly assigned to one of the seven experimental groups. The study adopted a between-subjects design. A pre-questionnaire on demographics was then completed by the participants. This was followed by the assessment of their initial levels of relaxation, happiness and fear, i.e. before any intervention. They were also asked to indicate their expectations of the emotions they would feel in the VR experience. This was done to allow assessment of potential biases resulting from participants’ existing knowledge of VR which could have influenced their *a priori* expectations of emotions.

**Fig 3:**
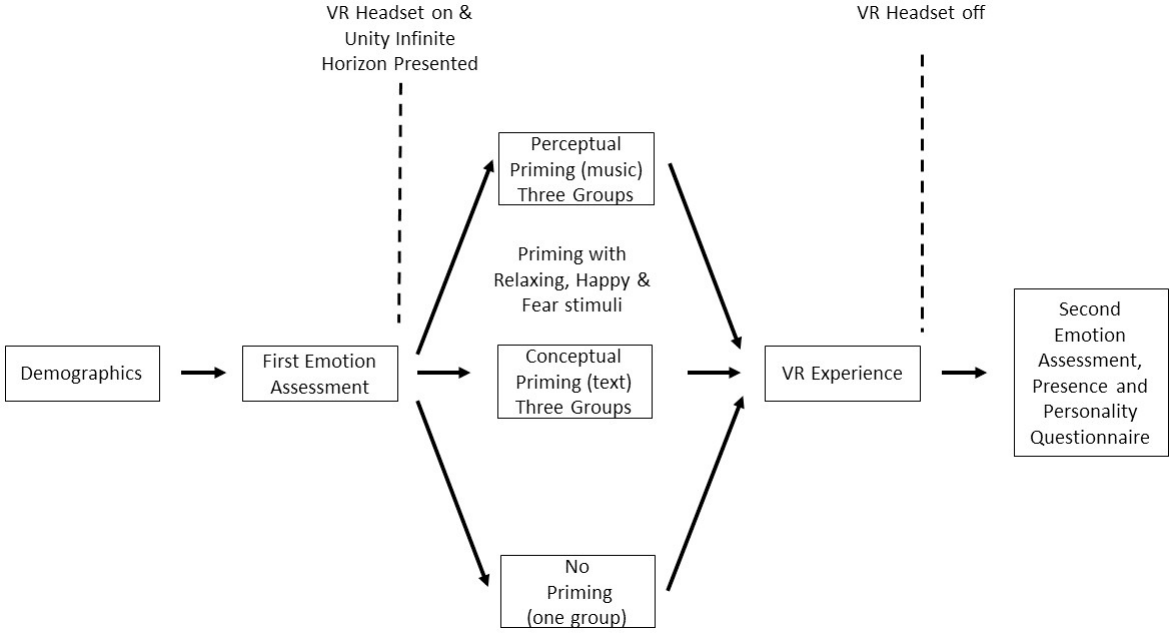
Visual representation of the study procedure in chronological order.

Next, participants were fitted with the Oculus Rift and listened to the priming material while a Unity default infinite horizon was played to control for visual input. The only difference between the seven experimental conditions was the auditory content that participants were exposed to. Participants in the three perceptual priming experimental conditions listened to music either eliciting relaxation, happiness or fear. The other three conceptual priming experimental groups, in contrast, listened to the priming material eliciting relaxation, happiness or fear. Participants in the control condition only experienced the same infinite horizon for the same amount of time, but did not receive any priming prior to the VR experience. The VR infinite horizon also served to familiarize the participants with the Oculus Rift head tracking and allowed them to accommodate to any VE-specific distance compression [93]. The latter manifests as an underestimation of distance in VR and may have the potential to affect the formation of spatial presence due to erroneous perception of distances between the user and features in the VE [26, 41]. For participants in the conceptual conditions, the review was played portraying the experience as being relaxing, happy or frightening respectively. For participants in the perceptual conditions, the music selected to induce the corresponding emotion was presented (see Fig 3).

After the priming phase, levels of felt and expected emotions were measured a second time for the three emotions, using the same questions as before. This second measurement of emotion expectations was conducted in order to test the effect of the priming interventions. Next, the ‘Senza Peso’ VR experience was presented. Before commencing the experience, participants were verbally encouraged to make use of the head tracking and look around as much as they liked and to anything in the VE capturing their attention. After this VR experience, the headset was removed and the levels of felt emotion were measured, followed by the battery of presence questionnaires. After the experimental phase, participants were administered the BFI via the Qualtrics platform and were debriefed.

### 4.9 Participants

A total of 126 participants (51 males, 75 females) over the age of 18 (mean age = 26.33, range = 18–65, SD = 8.71) were recruited via opportunity sampling. Each of the seven experimental groups consisted of 18 participants with counterbalanced numbers of males and females. Participants were randomly assigned to one of the experimental conditions. They were a mixture of undergraduate (39), masters (35) and PhD (19) students as well as university staff members (30) and others, who were university alumni (three). All participants had normal or corrected to normal vision and hearing. In terms of previous experience with VR, 42 declared they had ‘none at all’, 63 had ‘a little’, 17 had ‘a moderate amount’, while only one had ‘a lot’ and three ‘a great deal’. There was also no significant difference between the seven groups in terms of their age (*p* = .751), education (*p* = .916) and experience with VR (*p* = .426). No participants withdrew from the study before its end.

The sample size was calculated with an *a priori* power analysis for a multiple linear regression analysis, fixed model, by using G*Power 3.1 [94]. For the estimation we used a Cohen’s *f*^2^ of 0.15 (for a medium effect size), a level of power of 0.80, nine predictors (induced emotion, type of priming, the three measured emotions and five personality traits), and an *α*-level of 0.05. This analysis returned a total necessary sample size of 114, i.e., 16 participants per group. The study was approved by the Psychology Research Ethics Committee (PREC) of the University of Bath (ethics code: PREC 18–301). All participants were adults and gave written informed consent prior to participating and were debriefed accordingly at the end of the study. Participants were also informed they were free to withdraw from the study at any point, but none chose to do so.

## 5 Statistical analyses

Scores were computed for the three emotions by subtracting the felt emotion score (third measurement which was collected after the VR experience) from the predicted emotion score that had been recorded after the priming phase but before the VR experience (second measurement). These scores are referred to hereafter as the computed relaxation, happiness and fear scores. Negative scores represent an overestimation of emotion levels in the participant’s prediction, and positive scores represent an underestimation. The closer a computed score is to zero, the greater the match between the predicted emotion score and its corresponding experienced emotion score. The IPCM predicts that the closer the computed emotion scores are to zero, the higher the level of presence should be.

To verify our hypotheses, several statistical tests were considered. Due to the continuous covariates, we could not use simple ANOVAs, but had to employ either univariate ANCOVAs or multiple regressions. These two are statistically equivalent and would have yielded similar results. However, there was a larger number of continuous variables compared to categorical, which suggests that regression analysis would be more suited. Second, our continuous predictors were of primary interest rather than being covariates to control for, hence we preferred using a multiple regression analysis. We finally ran a comprehensive multiple regression analysis rather than separate analyses to address each hypothesis as we wanted to consider the possible interaction effects among all the different predictors, which could have been lost if running separate analyses.

In consequence, a total of five multiple linear regressions were carried out to test the effect of all predictors on the measures of presence, i.e. on spatial, social and self presence, on the presence question (Q2) from the questionnaire by Egan et al. [74] and on the UCL presence questionnaire score [87]. An ‘enter’-type selection process was used because we could not predict which predictors would create the best prediction equation. The predictors were the computed relaxation, happiness and fear scores, the five personality trait scores and the seven priming conditions: relaxation conceptual, relaxation perceptual, happiness conceptual, happiness perceptual, fear conceptual, fear perceptual and control. For conducted t-tests we report Cohen’s d as a measure of effect size.The analysis was conducted using the IBM SPSS Statistics (Version 28) software [95].

## 6 Results

For clarity, we will split the results section based on the predicted variables, namely the five measures of presence that were used, rather than per hypothesis. For each section we mention the implications of the presented results for our hypotheses. At the end of the section, we discuss the extent to which we can accept each hypothesis.

### 6.1 Spatial presence

For spatial presence, as expected, no significant regression was found (*F*(3, 111) = 1.153, *p* = .321). Only agreeability acted as a significant predictor. This yielded a significant regression equation (*F*(1, 124) = 7.044, *p* = .009), with an *R*^2^ of.054. Spatial presence was equal to 5.328 +.051 (*p* = .009) (computed fear score). This suggests that computed emotion scores cannot predict spatial presence; cf. social and self presence in **H1**. Agreeability predicting spatial presence partially supports **H4**.

### 6.2 Social presence

In the case of social presence, a significant regression equation was found (*F*(14, 111) = 1.80, *p* = .046), with an *R*^2^of.185. Two significantly contributing factors were found, namely the computed fear score (*p* = .026) and agreeableness score (*p* = .034). Next, we examined whether there was any interaction effect between fear and agreeableness scores. To correct for collinearity between the interaction scores (calculated as the product of computed fear scores and agreeableness scores) and the original scores for fear and agreeableness, we centered the data for all these predictors and checked for correlations. No correlation was found among the centered predictors (*p* > = .338), thus showing lack of multicollinearity. A regression with agreeableness, computed fear score and the interaction variable was run. This yielded a significant regression equation (*F*(3, 122) = 5.778, *p* = .001), with an *R*^2^ of.124. Participants’ predicted social presence was equal to 4.732 +.169 (*p* = .006) (computed fear score) +.073 (*p* = .002) (agreeability score). No interaction effects were found between agreeableness and fear scores (*p* = .894). Computed fear scores predicting social presence partially supports **H1** and **H2**. The lack of an effect of perceptual or conceptual priming means that **H3** is rejected. **H4** is partially supported as only agreeableness predicted social presence, but not openness and introversion.

### 6.3 Self presence

For self presence, a non-significant regression equation was found (*F*(14, 111) = 1.127, *p* = .343), with an *R*^2^of.124. However, two significantly contributing factors were found, namely computed happiness scores (*p* = .036) and computed fear scores (*p* = .036). This prompted a regression analysis with just these two predictors, as for social presence. No interaction variable was calculated between happiness and fear computed scores as we did not hypothesize any interaction between them. The analysis showed a significant regression equation (*F*(2, 123) = 3.213, *p* =.044), with an *R*^2^ of.050. Participants’ predicted self presence was equal to 4.909 +.177 (*p* =.041) (computed happiness score) +.136 (*p* =.023) (computed fear score). These results suggest that self presence is predicted by both computed fear and computed happiness scores, unlike spatial and social presence. No personality traits predicted self presence. The computed happiness and fear scores predicting self presence supports **H1**. The fact that computed happiness and computed fear, but not computed relaxation, predicted self presence supports **H2**. The lack of an effect of perceptual or conceptual priming means that **H3** is rejected. **H4** is not supported as no personality traits predicted self presence.

### 6.4 Overall presence

**Fig 4:**
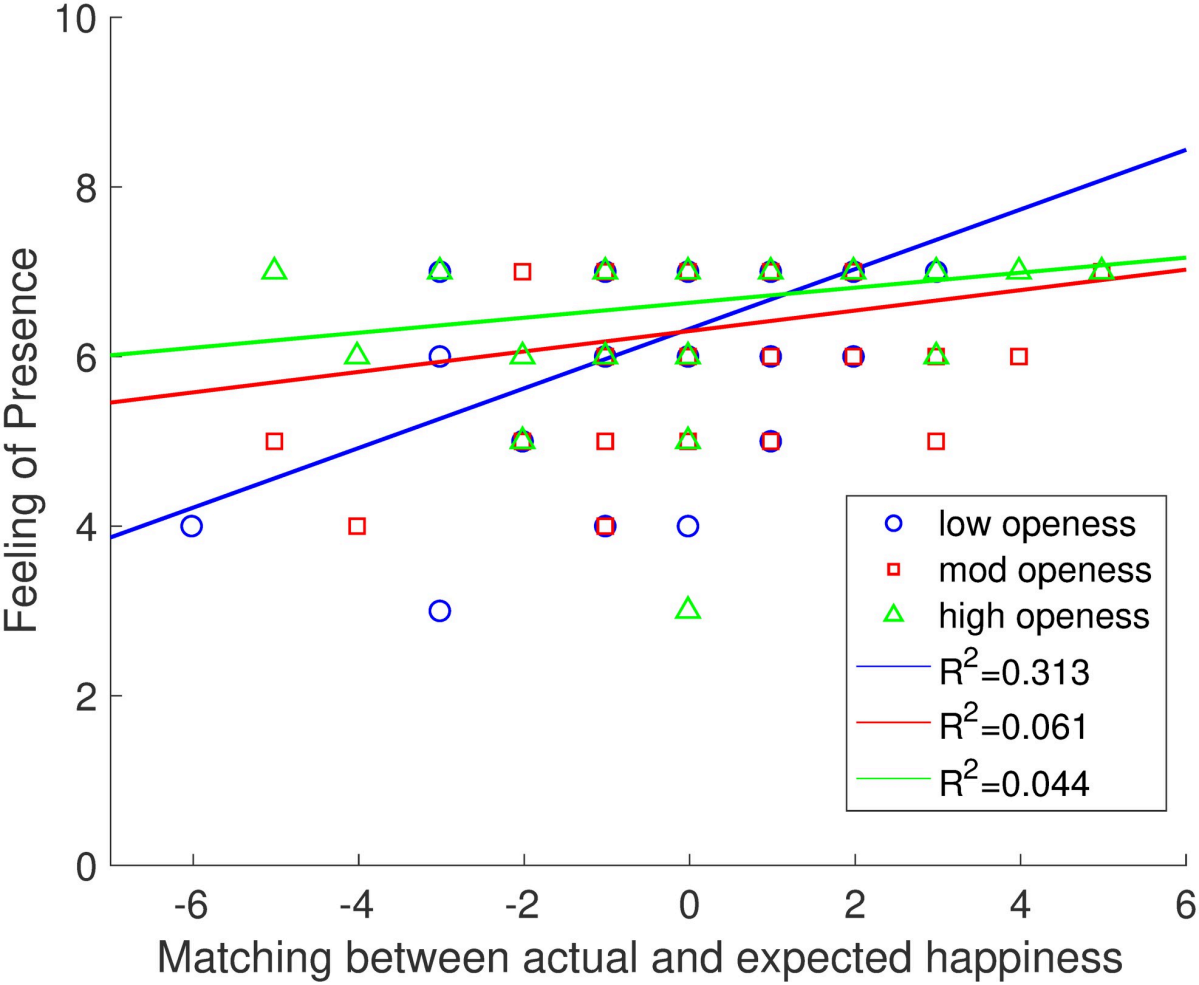
Interaction between openness and happiness scores when predicting presence. The blue line represents the best linear fit to presence scores for the low openness group. The blue circles represent that data points for the low openness group. The red line represents the best linear fit to presence scores for the medium openness group. The red squares represent the data points for the medium openness group. The green line represents the best linear fit to presence scores for the high openness group. The green triangles represent the data points for the high openness group. R2 values for the different levels of openness can be found in the legend.

For presence as measured by Q2 in Egan et al.’s questionnaire [74], we found a significant regression equation (*F*(14, 111) = 2.768, *p* <.001), with an *R*^2^ of.259. Two significant contributors were found for the model, namely computed happiness score (*p* =.046) and openness (*p* =.033). Next, we examined whether there was any interaction effect between happiness and openness scores. To correct for collinearity between the interaction scores (calculated as the product of happiness scores and openness scores) and the original scores for happiness and openness, we centered the data for all these predictors and checked for correlations. No correlation was found among the centered predictors (*p* > =.161), indicating a lack of multicollinearity. A significant regression equation was found (*F*(3, 122) = 9.252, *p* <.001), with an *R*^2^ of.259. Participants’ presence was equal to 6.428 +.028 (*p* = 0.49) (openness score) +.166 (*p* <.001) (computed happiness score)—.018 (*p* =.020) (openness and happiness score interaction variable). An interaction was found between computed openness and happiness scores (see Fig 4). The regression lines represent the data for low, medium and high openness. The higher *R*^2^ value obtained for people scoring low on openness indicates that this group was more susceptible to differences between predicted and felt emotions. For those scoring medium or high openness this effect was not so pronounced, suggesting more sensitivity to large discrepancies. Results for Q2 suggest that happiness is a strong predictor of presence in general. Furthermore, the interaction with openness shows that personality traits can moderate not only presence, but the relationship between presence and emotion. The fact that computed happiness, but not computed relaxation, predicted Overall presence partially supports **H2**. **H4** is also partially supported because openness predicted Overall presence.

A non-significant regression equation was found for the UCL presence questionnaire measure (*F*(14, 111) = 1.274, *p* =.235), with an *R*^2^ of.138. Only the computed fear scores acted as a significant predictor, which partially supports **H2**. This yielded a significant regression equation (*F*(1, 124) = 5.228, *p* =.024), with an *R*^2^ of.040. Presence as measured by the UCL questionnaire was equal to 4.887 +.088 (*p* =.024) (computed fear score), suggesting that computed fear scores predicted presence. The UCL presence questionnaire thus showed different results to presence as assessed by Egan et al.’s Q2 (see Table 2). More precisely, computed fear scores seem to be a good predictor of presence as measured by the UCL questionnaire, and not computed happiness, as was the case with presence measured via Q2.

**Table 2:** Table showing the regression results for the presence measures.

Variable	*F*	*p*	*R* ^2^
Spatial Presence	1.153	.321	.167
Social Presence	1.800	.046	.185
Self Presence	1.127	.343	.124
UCL Presence	1.274	.235	.138
Overall Presence	2.768	.001	.259

### 6.5 Testing IPCM predictions

**Fig 5:**
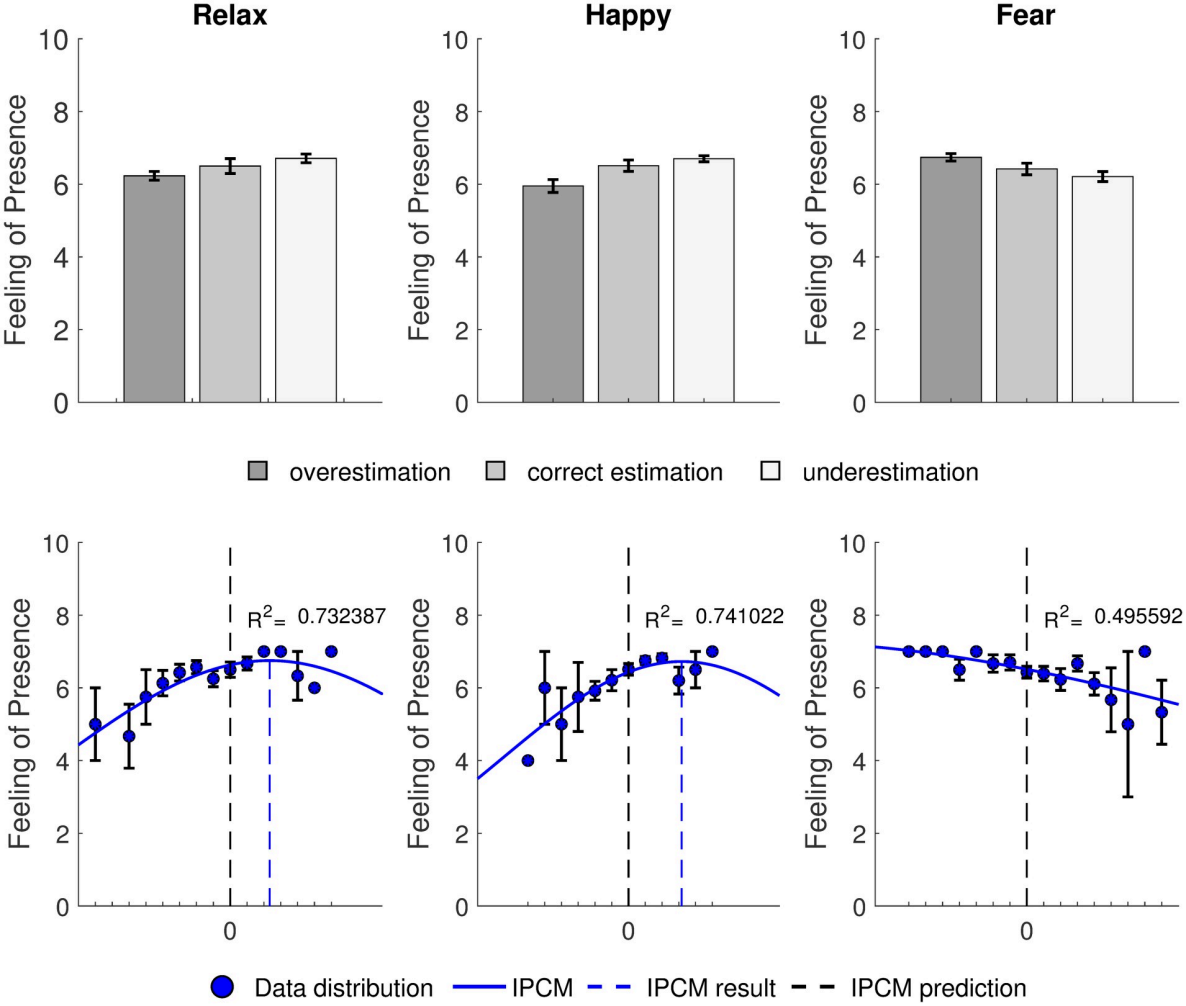
Graphs at the top show mean scores of presence as measured by Q2 for participants who overestimated, estimated correctly and underestimated levels of relaxation, happiness and fear. Graphs at the bottom represent mean distributions of the gaussian psychometric functions for presence, as measured by Q2 for relaxation, happiness and fear. The solid blue line represents the line of best fit for these data points. It represents the normal distribution fitted to the data as predicted by the IPCM. The dashed blue line represents the actual peak in presence and the black dashed line represents the peak in presence as hypothesized by the IPCM.

We investigated whether overestimating and underestimating emotion equally lead to a decrease in presence, as suggested by Seth et al. in their model [3]. For this analysis we chose Egan et al.’s Q2 since it represented the most general measure of presence [74]. We ran Bonferroni corrected independent samples t-tests between those who underpredicted, predicted correctly, and overpredicted emotion intensity. For Q2, we found that the underpredicting group (*Mean* = 5.951, *SD* = 1.139) achieved significantly less presence than those who overpredicted emotion (*Mean* = 6.708, *SD* =.581); *t*(87) = −4.032, *p* <.001, *d* = −.319. There was no significant difference between the underpredicting group and the group who predicted correctly (*Mean* = 6.513, *SD* =.960), or between the group who predicted correctly and the overpredicting group (*p* > =.066). Results were similar for fear, in that the only significant difference found was between the underpredicting group (*Mean* = 6.7419, *SD* =.575) and the overpredicting group (*Mean* = 6.216, *SD* = 1.075); *t*(89) = 2.535, *p* =.039, *d* =.011. For relaxation, no differences were found between any of the groups (*p* > =.051) (see Fig 5). These results further support the IPCM and **H1**.

### 6.6 Emotion intensity

Paired-samples t-tests were conducted to compare the felt emotion scores (third measurement) for relaxation, happiness and fear for the entire sample size. There was a significant difference between the scores for relaxation (*Mean* = 5.73, *SD* = 2.37) and happiness (*Mean* = 7.23, *SD* = 1.99;*t*(125) = −7.387, *p* <.001, *d* = −.658, between relaxation and fear (*Mean* = 2.68, *SD* = 2.47); *t*(125) = 7.923, *p* <.001, *d* =.706, and between happiness and fear; *t*(125) = 13.631, *p* <.001, *d* = 1.214. Thus, happiness scores were overall highest, followed by relaxation and lastly by fear.

### 6.7 Discomfort and emotion/presence reports

Because post-experience emotion scores are emotional appraisals of the VR app, we took into account that negative experience due to cybersickness, which can cause sensations of dizziness or nausea, could impact these scores. Discomfort could be due to motion sickness, or general dissatisfaction with the system hardware [50, 74]. To exclude this possibility we ran a correlation analysis between presence scores and general enjoyment as measured by Q9 in Egan’s [74] questionnaire which referred to general discomfort while using the system (“I did not feel any discomfort while using the application”). Results showed no significant correlation between discomfort and reported levels of emotion during the VR experience for relaxation, happiness or fear (*p* >.051). No significant correlation was found between general comfort and presence measured by any of our measures (*p* >.382). Hence, physical discomfort or lack of satisfaction with the VR hardware did not affect our results.

### 6.8 Summary of hypotheses validation

**Fig 6:**
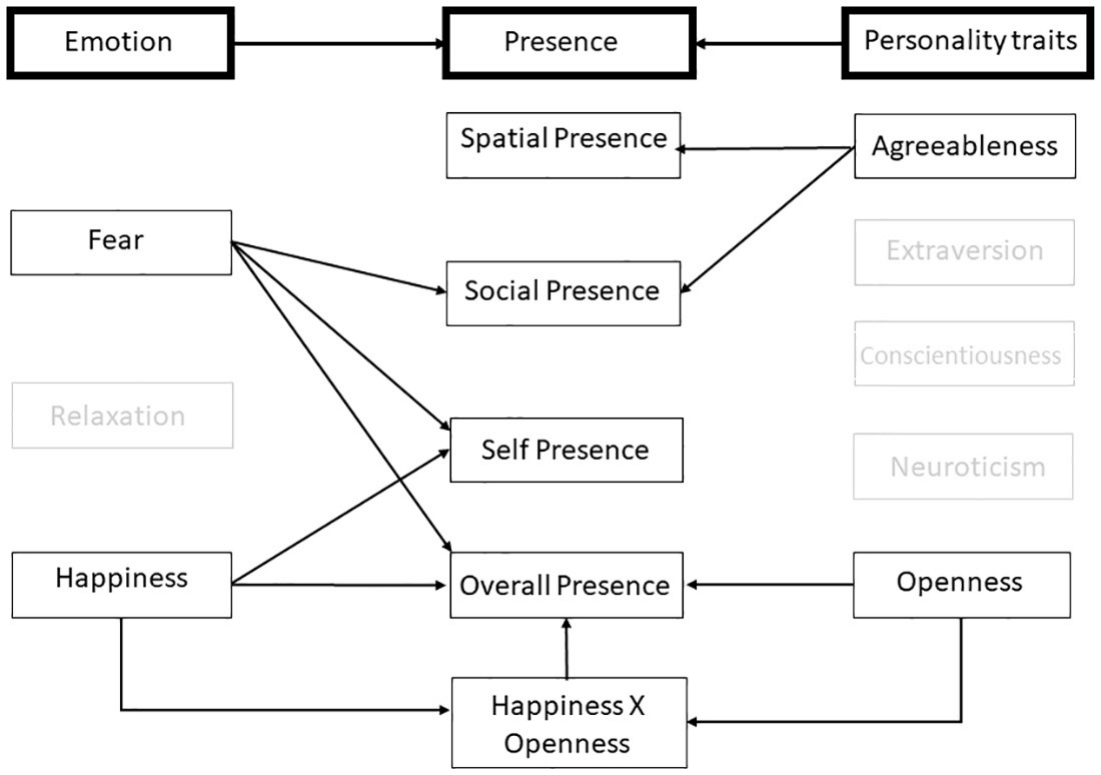
Graphical representation of the revised IPCM model, as informed by the regression results. The left row presents the three emotions tested. The middle row presents the types of presence that were verified and interactions between factors impacting overall presence. The right column presents the BFI [72] personality traits tested. Arrows signify significant interactions and items that are in grey did not show any significant impact on presence measures.

Our results showed that, as hypothesized in **H1**, the IPCM predicted the formation of social and self presence but not spatial presence. Moreover, **H2** was supported because the IPCM predicted presence based on computed happiness and computed fear but not computed relaxation. The lack of an effect of perceptual or conceptual priming on any of the emotions or components of presence means that **H3** is rejected. Lastly, results showed that some personality traits, namely openness and agreeableness moderate the relationship between emotion and presence, and so we partially accept **H4**. A schematic presenting the significant interactions found can be seen in Fig 6.

## 7 Discussion

Our results show for the first time that the IPCM can explain the formation of presence in VR environments. As expected, the IPCM predicted the formation of social and self-presence, but not spatial presence, supporting H1. The model can predict presence based on computed happiness and fear scores, but not relaxation scores, depending on the measure of presence which supports H2. H3 was rejected since priming via the perceptual and conceptual pathways did not yield any changes in presence. Another novel finding is that some personality traits influenced the relationship between emotion and presence, supporting H4. These results prompt a proposed revision to the IPCM, presented below.

The IPCM was found to predict social presence but only fear was a significant predictor. This may be explained through an evolutionary typology of fear. Mayr [96] proposed that social phobia is a major evolutionary derived trait that is inherent in humans. Fear is also the most prioritized emotion in interactions with others due to its function in surviving threats and triggering fight or flight responses [97]. Thus, as hypothesized in H2, highly-arousing emotions have a greater impact on presence compared to low-arousal emotions. This pattern of results suggests that the IPCM applies to the high-arousal emotion that is most prominent in a given VE.

This suggestion is supported by the other results. Self presence was predicted by both the computed happiness and computed fear scores, while relaxation did not yield significance. It seems, therefore, that users drew on high-arousal emotions when constructing self presence. Ratan and Hasler [98] created a standardized measure of self presence and suggested that arousal may be a core determinant of it. Our results not only support this hypothesis but also offer new insights into the relationship between arousal and self presence. Our findings suggest that self presence is determined by the matching of predicted and actually felt high-arousal emotions but not low-arousal ones. Thus, low-arousal emotions such as relaxation do not contribute to the creation of presence. Future studies could investigate the moderation effect of arousal by looking at relevant physiological measures (e.g. skin conductance and heart rate).

Spatial presence refers to the ability of the user to locate themselves relative to features in the environment [27]. It is dependent on geometric computations and distance perception which are not facilitated by users’ emotions but rather by technical features of the VR system [38] and by the human perceptual system [93]. Hence, the lack of an effect of emotions on spatial presence was expected. Previous studies treating presence as an unitary concept have found that emotions may impact its formation (e.g. [23, 80]). Based on our findings we believe that the effect of emotions on presence formation is specific to social and self-presence rather than to the sense of presence overall, which could explain the previous contradicting results on the role of emotion on presence [23, 24, 80, 99].

When Overall presence was measured using Egan et al.’s Q2 [74], we found that it was predicted by the computed scores of happiness but not those of relaxation or fear. This suggests that when assessing Overall presence, users consider how satisfied or happy they were with the experience. Satisfaction and happiness have been shown to be highly valued by users in the context of VR [42]. Thus, when presented with a single question about how present they had felt, users may simply have drawn on their satisfaction or disappointment with the VR experience. However, the UCL questionnaire showed the opposite result to Egan’s question in that the model applied to fear but not to happiness scores. Hence, while the IPCM seems to work well for different measures of presence the type of emotion examined may determine how well this model can explain the formation of presence. Hence, the influence of types of emotion and level of arousal should be included in the model to increase generalisation.

When comparing presence scores between participants who underpredicted and those who overpredicted levels of happiness, we found that the underpredicting group achieved higher presence. In other words, participants who felt more happiness than they had predicted in the VR experience achieved higher presence than those who overestimated the level of happiness they would feel. An inverse result was found for fear in that overpredicting fear led to higher presence compared to underpredicting it. Thus, it seems that unexpectedly high intensity emotions are detrimental to presence if they are negative but can be beneficial if positive. In other words, the level of presence seems to be linked to the level of positive emotional experience, which is not unexpected. No effect was found for relaxation, which strengthens the idea that low-arousal emotions are not heavily weighted in determining presence. This pattern of results is inconsistent with the IPCM which predicts a uniform decrease in presence independent of whether participants underestimate or overestimate emotion.

Openness, or openness to experience as it is often referred to, significantly predicted presence as assessed by the question used previously by Egan et al. [74]. We found that more open individuals achieve higher levels of presence, confirming previous findings [78]. In addition, openness moderated the relationship between computed happiness and presence, in that the more open individuals were, the more tolerance they showed to large discrepancies between predicted and felt emotions. Hence, the correlation between computed happiness scores and presence is weaker for moderate to high openness as this trait works as a buffer in the relationship between these two variables.

This finding supports the mechanism proposed by the IPCM. Seth et al. [3] suggest that users must suppress these discrepancies between predicted and felt emotion to achieve presence; and it is reasonable to conclude that people who are more open to experiences are more likely to do so. That is, even though the VR experience was not as happiness inducing as they expected, more open individuals ignored this discrepancy and consequently achieve higher presence compared to those that could not ignore such discrepancy. This finding offers support not only to the IPCM but also to the idea that presence is significantly determined not just by features of the VR system but also by user characteristics.

Agreeability was found to be a predictor for social presence but there was no interaction between agreeability and computed fear. Hence, the effects that agreeability and computed fear had on social presence were independent of each other. The finding that more agreeable people seem to achieve higher levels of social presence is not surprising given that social presence itself is defined as the impression of ‘being amongst others’ or, as found by Shin [42], of being connected with other social users present in the VE. People who score higher on agreeableness are known to be more easy-going and friendly in social situations [84]. Agreeability also influenced spatial presence in that participants scoring higher on this trait showed higher levels of spatial presence. This result is identical with the findings of Sacau et al. [84] who showed that, of the BFI personality traits, only agreeableness was a predictor of spatial presence. They suggest that this is due to more agreeable people showing higher empathy, which can manifest even to non-living objects [100]. This in turn could lead to a better spatial understanding of where these objects are relative to the user [84].

Similarly to the findings for emotion, not all personality traits consistently moderated or predicted all types of presence. In line with our findings, Kober and Neuper [78] found that personality traits showed heterogeneous correlations with presence, depending on the presence questionnaire that was utilized. They suggested that because these questionnaires have different theoretical backgrounds, they measure different facets of presence, which leads to inconclusive correlations with personality traits. Our results reinforce the need for more standardized instruments that can reliably and independently assess various aspects of presence. Future research should conduct more focused studies investigating those factors which were identified here as involved in the creation of presence. Such a design would benefit from structural equation modelling analysis, which would offer a clearer picture of the extent to which each factor contributes to the formation of presence (for an example investigating presence, see [76]).

Based on our findings, we propose several refinements to the IPCM. First, the model should not treat presence as a unitary concept but rather should consider its components separately. Our findings suggest that this adapted model could be effectively applied to understanding the formation of social presence and self presence but not spatial presence. Secondly, the model should include the role of emotions and weight them with respect to their influence on presence. This should be done according to the level of arousal of the emotions elicited by the VE as well as to the type of emotion (i.e., positive and negative). For example, the higher the level of arousal, the greater impact a specific emotion should have on presence. And the direction (not only the magnitude) of the mismatch should be considered depending on the valence of the emotion when predicting presence. Finally, the model should take into account that users’ personality, in particular openness to experience, moderates the degree to which emotions determine presence.

### 7.1 Limitations

Although not affecting our results for H1, H2 and H4, priming participants with conceptual or perceptual stimuli did not show an effect, thus not supporting H3. One possible explanation for this is our use of audio-only stimuli for the priming. VR is a rich, multisensory medium and thus the priming we attempted via a unisensory stimulus may not have had a large effect. Future research could attempt priming using multisensory stimuli, preferably presented in VR. This could clarify whether the use of unisensory priming was indeed the reason why priming did not succeed. Still, it is worth noting that because the computed emotion scores were calculated using the scores collected after the priming phase and those collected after the VR experience, the lack of a priming effect has no impact on our other findings.

An important limitation related to social presence in this study is the lack of user interaction with the virtual avatars present in the experience. This is because social presence is not only determined by the mere presence of other sentient beings, but also interactions with them which would lead to more complex inferences about their thoughts or emotions [44]. This means that in the present study, social presence may not have been felt the same way as it would be in VR applications which do facilitate interaction with other social beings within the VE [101]. This lack of interaction was intentional, so as to not introduce potential confounding variables to the design due to how users chose to interact or not with the VE. Still, this does not mean that the present results on social presence are not valid, but that they strictly apply to VEs where there exists no interaction with other social agents. Further research should focus on social presence and employ more sophisticated VEs where such interactions would be possible.

Using self-report measures for both emotion and presence could also be a limitation as judging the intensity of felt emotions could be considered when reporting presence and vice-versa. As observed by IJsselsteijn [102] an emotional response towards the VE (e.g. a fear or happy response) can be interpreted by the participant as a sign of a higher level of presence, possibly due to the existence of specific internal feedback mechanisms.

Measuring either emotions or presence via an objective measure could be a solution and seems promising, but has so far been unsuccessful in the context of VR presence [74, 79]. Additionally, while such measures may indeed measure arousal, it is impossible to estimate emotional valence (e.g. whether the user is happy or fearful) from these measures. Nonetheless, self report measures themselves pose limitations, such as being prone to demand characteristics; which do not affect physiological measures to the same extent [103]. Demand characteristics could be especially relevant if users are primed with conceptual material, like we did in the present study. This means that perhaps physiological measures could be used in conjunction with self report ones to validate obtained emotion scores.

Apart from the UCL Presence Questionnaire [73], the present study used custom, individual items for the three types of presence examined and a single item from Egan’s [74] study to measure overall presence. Future studies should use more recent and well-validated measures which were not available at the time of our study, such as the Multimodal Presence Scale (MPS) [104], which has subscales for spatial, social and self presence.

## 8 Conclusion

Previous studies have often focused on the formation of spatial presence within VR, but the processes that lead to social and self-presence remain relatively under researched. Furthermore, the role played by emotions in the formation of these components of presence has been largely unexplored. Here we show that an interoceptive predictive coding model can be applied to VR to understand how presence is created and the role that emotions play in this process. We show that the model does apply for a selection of highly-arousing emotions and that the results can depend on how presence is measured. We propose that, depending on the type of presence assessed and VR content presented, highly-arousing emotions may be prioritized when creating presence.

We have also shown that users’ expectations of the emotions they will experience in a VR application can be of crucial importance in the creation of presence. In turn, users’ expectations of high-arousal emotions such as happiness and fear may be manipulated to enhance presence in VEs. Overprediction and underprediction of such emotions do not have symmetric effects on presence, and we propose that the IPCM should be refined to account for this asymmetry. We have also shown how some personality factors can moderate the creation of presence. Incorporating this range of findings, we propose the creation of a refined, integrated model of presence that encompasses predictive coding of high-arousal emotions and accounts for individual differences.

## Supporting information

S1 Data (XLSX)
